# Prognosis research ideally should measure time-varying predictors at their intended moment of use

**DOI:** 10.1186/s41512-016-0006-6

**Published:** 2017-02-08

**Authors:** Rebecca Whittle, Kara-Louise Royle, Kelvin P. Jordan, Richard D. Riley, Christian D. Mallen, George Peat

**Affiliations:** Arthritis Research UK Primary Care Centre, Research Institute for Primary Care & Health Sciences, Keele University, Keele, Staffordshire ST5 5BG UK

**Keywords:** Primary health care, Prognosis, Multivariable prediction models, Musculoskeletal pain, Point of care, Time-varying predictors, Bias

## Abstract

**Background:**

Prognosis research studies (e.g. those deriving prognostic models or examining potential predictors of outcome) often collect information on time-varying predictors *after* their intended moment of use, sometimes using a measurement method different to that which would be used. We aimed to illustrate how estimates of predictor-outcome associations and prognostic model performance obtained from such studies may differ to those at the earlier, intended moment of use.

**Methods:**

We analysed data from two primary care cohorts of patients consulting for non-inflammatory musculoskeletal conditions: the Prognostic Research Study (PROG-RES: *n* = 296, aged >50 years) and the Primary care Osteoarthritis Screening Trial (POST: *n* = 756, >45 years). Both cohorts had collected comparable information on a potentially important time-varying predictor (current pain intensity: 0–10 numerical rating scale), other predictors (age, gender, practice) and outcome (patient-perceived non-recovery at 6 months). Using logistic regression models, we compared the direction and magnitude of predictor-outcome associations and model performance measures under two scenarios: (i) current pain intensity ascertained by the treating general practitioner in the consultation (the intended moment of use) and (ii) current pain intensity ascertained by a questionnaire mailed several days after the consultation.

**Results:**

In both cohorts, the predictor-outcome association was substantially weaker for pain measured at the consultation (OR (95% CI): PROG-RES 1.06 (0.95, 1.18); POST 1.04 (0.96, 1.12)) than for pain measured in the questionnaire (PROG-RES 1.34 (1.20, 1.48); POST 1.26 (1.18, 1.34)). The *c*-statistic of the multivariable model was lower when pain was measured at the consultation (*c*-statistic (95% CI): PROG-RES 0.57 (0.51, 0.64); POST 0.66 (0.62, 0.70)) than when pain was measured in the questionnaire (PROG-RES 0.69 (0.63, 0.75); POST 0.72 (0.68, 0.76)), reflecting the lower OR for pain at the consultation.

**Conclusions:**

Prognostic research studies ideally should measure time-varying predictors at their intended moment of use and using the intended measurement method. Otherwise, they may produce substantially different estimates of predictor-outcome associations and model performance. Researchers should report when, how and where predictors were measured and identify any significant departures from their intended use that may limit the applicability of findings in practice.

**Trial registration:**

The protocol for the PROG-RES cohort data collection and primary analysis has been published in an open-access journal (Mallen et al., BMC Musculoskelet Disord 7:84, 2006). The POST trial was registered (ISRCTN40721988; date of registration: 21 June 2011; date of enrolment of the first participant: 3 October 2011) and had a pre-specified protocol covering primary analysis. There was no published protocol for the current secondary analyses presented in this manuscript.

## Background

Prognosis research studies are used to help summarise and predict future outcomes in patients with a particular disease or health condition [1]. In particular, many studies which examine potential predictors (prognostic factors) of outcome risk [2] and/or develop a prognostic model containing multiple predictors for individualised risk prediction [3] are published each year. Prognostic models are intended “to assist clinicians with their prediction of a patient’s future outcome and to enhance informed decision making with the patient” [4]. Predictions from these models should have optimal performance at the time that they are practically implemented—the “intended moment of using the model” [5]. The TRIPOD (Transparent Reporting of a multivariable prediction model for Individual Prognosis or Diagnosis) statement recommends to clearly define when the predictors used in the development of the model were measured [6] and states that “all predictors should be measured before or at the study time origin and known at the intended moment the model is intended to be used” [7]. In the context of primary care, this will typically be at the point of care—the primary care consultation. For a range of practical and ethical reasons, researchers may design prognosis research studies that collect predictor information *after* the intended moment of use. For example, one approach commonly used in prognosis studies of recurrent and long-term conditions presenting to primary care is for information on predictors (such as pain intensity) to be ascertained by mailed self-complete questionnaires, or personal interview and examination in research clinics several days *after* their index consultation (e.g. [8–14]). This approach offers several advantages, as it facilitates: 1) a wider range of predictor information to be collected than would be possible within the time-constrained primary care consultation, 2) greater standardisation of data collection procedures, 3) a “cooling off period” between being informed about the study at the point of care, and consenting to provide information on potential predictors that would not be considered part of routine care. However, this practice also carries potential limitations when the measured values of the predictors included in these studies are time-dependent and particularly when they may additionally be sensitive to the choice of measurement, mode of administration and other contextual influences on participants’ responses [15–17]. In these circumstances, estimates of predictor-outcome associations and prognostic model performance obtained from the study may be systematically different (biased) from those that would have been observed had those predictors been measured at the point of care. This problem is what is referred to as indirectness in the GRADE guidelines [18], the effect of which could be assessed in a particular prognostic model if external validation was performed in a setting and timeframe the same as when the model would be used in practice, as recommended in the REMARK guidelines [19].

The aim of this study was to illustrate this concern using a real example, showing how using a measure recorded shortly after a patient’s index consultation to develop a prediction model can provide misleading prediction estimates if used during this index consultation. Developing a prediction model intended to be implemented in practice is not the purpose of this study. We compare the direction and magnitude of predictor-outcome associations and also the differences in the Akaike’s information criterion (AIC) and *c*-statistic of a multivariable prognostic model, under two scenarios: firstly, using a time-varying predictor of interest, ascertained by the treating physician at the point of care (i.e. at the intended moment of use), and secondly, using the same predictor, but ascertained by a self-complete questionnaire mailed several days after the point of care. Our predictor of interest is current pain intensity in patients presenting to primary care with non-inflammatory musculoskeletal disorders, which has previously been found to be a predictor of unfavourable episode outcomes in several previous primary care studies [20].

## Methods

We undertook secondary analyses of two primary care longitudinal datasets: the Prognosis Research Study (PROG-RES), an observational study [21], and the Primary care Osteoarthritis Screening Trial (POST) (ISRCTN40721988), a cluster randomised trial. PROG-RES focussed on consultation for non-inflammatory musculoskeletal pain and POST on peripheral joint osteoarthritis. Both studies had included a brief, standardised assessment of predictors during the consultation (point of care) by the treating general practitioner (GP) which they recorded on the practice computer. The studies had similar patient populations, recruitment procedures, and measurement of predictors and outcome, thereby allowing us to observe whether similar findings were present within the two comparable studies (Table 1).

**Table 1:** Design and sample characteristics of the two questionnaires

	PROG-RES	POST
Design	Prospective observational cohort	Cluster RCT
Registration	(Protocol [21])	Current Controlled Trials ISRCTN40721988
Intervention	Usual care	I: ultra-brief screening questions for anxiety and depression + pain intensity measurement C: screen for pain intensity
Setting	5 general practices in North Staffordshire, England	45 general practices in West Midlands, England
Period of recruitment	Sep 2006–Apr 2007	Sep 2011–Nov 2012
Inclusion criteria	Consecutive patients aged 50+ years consulting for non-inflammatory musculoskeletal pain	Consecutive patients aged 45+ years consulting for suspected or diagnosed peripheral joint osteoarthritis
Exclusion criteria	Vulnerable patient (e.g. diagnosed with dementia); recent trauma associated with significant injury; inflammatory arthropathy	Vulnerable patient (e.g. diagnosed with a terminal illness); nursing home resident; recent trauma associated with significant injury; inflammatory arthropathy, crystal disease, SpA, PMR
Data collection points^a^	*In GP consultation (point of care)*, *post-consultation questionnaire*, 3 months, *6 months*, 12 months, 24 months, 36 months	*In GP consultation (point of care)*, *post-consultation questionnaire*, 3 months, *6 months*, 12 months
Candidate predictor of interest	Current pain intensity (0–10 NRS [23])	
Timing of predictor measurement	1. Point of care 2. Post-consultation questionnaire	
Outcome of interest	Patient global rating of change at 6 months (completely recovered/much improved/improved vs same/worse/much worse [22])	
Participants eligible for inclusion in main analyses	296	756
Age (years): mean (SD)	64.8 (9.8)	65.8 (9.9)
Male: no. (%)	120 (40.5)	339 (44.8)
Current pain intensity at point of care (0–10): mean (SD)	5.9 (2.2)	6.2 (2.1)
Current pain intensity in questionnaire (0–10): mean (SD)	5.5 (2.6)	5.3 (2.6)
Interval between point of care and return of questionnaire (days): median (IQR; range)	17 (13, 27; 6–75)	21 (16, 30; 3–81)
Unfavourable outcome at 6 months: no. (%)	144 (48.7)	412 (54.5)

*Abbreviations*: *C* control, *GP* general practitioner, *I* intervention, *IQR* inter-quartile range, *NRS* numerical rating scale, *PMR* polymyalgia rheumatica, *POST* Primary care Osteoarthritis Screening Trial, PROG-RES Prognostic Research Study, *RCT* randomised controlled trial, *SD* standard deviation, *SpA* spondyloarthritis

^a^Data collection points indicated in italics are the collection points used for this analysis

The outcome of interest in the current study was the self-reported patient global rating of change recorded in the 6-month post-consultation questionnaire. The categorical responses were dichotomised into having experienced a favourable outcome (completely recovered, much improved or improved) or an unfavourable outcome (same, worse or much worse) [22].

Our key interest was the predictor-outcome association between an unfavourable outcome at 6 months and current pain intensity (0–10 numerical rating scale (NRS); 0 = no pain [23]). Pain intensity is a time-varying predictor, and we compared its association with an unfavourable outcome on two occasions: (i) at the point of care as recorded by the GP and (ii) recorded in a questionnaire by the patient sent within the week following point of care. Although the questionnaire was mailed within the week after the patient’s first visit to their GP, in both studies over a quarter of the questionnaires were returned at least a month after their consultation.

In both POST and PROG-RES, the post-consultation questionnaires and the instructions to GPs measured current pain intensity in the same standardised format with the same anchors: “How would you rate your pain on a 0–10 scale at the **present time**, that is **right now**, where 0 is ‘no pain’ and 10 is ‘pain as bad as could be’?”

Participants were eligible for inclusion in the current analyses if they had returned their questionnaire, consented to the use of medical records (such that their point of care information was available), and were successfully followed up at 6 months.

### Statistical analysis: predictor-outcome associations

Logistic regression models were fitted to estimate the adjusted predictor-outcome association between an unfavourable outcome at 6 months and pain intensity rating when recorded (i) at the point of care (i.e. intended point of using the prognostic results) and then (ii) in the questionnaire. Pain intensity was always included as a continuous variable, and its association with outcome was always included as a linear term. Adjustment factors within all of the models were age (as a linear term), gender and general practice. Only patients with complete predictor information at the point of care and the questionnaire, with outcome information available at 6 months, were included to ensure all analyses were comparable. Within the POST dataset, the models also included treatment arm as an additional adjustment factor, to account for any differences between the treatment and control groups within the study. The adjusted predictor-outcome association estimates (odds ratios (OR)) and 95% confidence intervals (CI) from the point of care model were compared with those from the questionnaire model, for each of PROG-RES and POST datasets separately. Although general practice was modelled as fixed effects, the same pattern of findings was observed when fitting general practices as random effects.

### Statistical analysis: prognostic model performance

Next, each of the logistic regression models fitted was considered as a prognostic model, such that they were to be (hypothetically) used for predicting individual outcome risk in new individuals. This allowed us to focus on their overall predictive performance and in particular to compare the performance of the models fitted at the point of care with the models fitted using the questionnaire information. The performance measures examined were the Akaike’s information criterion (AIC) and discrimination.

The AIC measures the relative goodness of fit of a model, considering both the statistical goodness of fit and the number of parameters used. The formula for the AIC is AIC = 2*K* − 2 ln(likelihood), where *K* is the number of parameters in the model and ln is the natural logarithm. The model with the lowest AIC is the preferred model, but as a rule of thumb, two models are essentially equivalent if the difference in their AICs is less than 3 units (when the sample size is greater than 256) [24].

We measured discrimination by the concordance index (*c*-statistic) [25], which is the ability of a model to differentiate between those who do or do not experience the outcome of interest; in this case, it is the ability of the model to differentiate between those who do or do not experience an unfavourable outcome at 6 months. The *c*-statistic is the probability that for any randomly selected pair of individuals, one with an unfavourable outcome and one without, the model assigns a higher probability to the individual with the unfavourable outcome. For logistic regression models, as used in this study, the *c*-statistic is identical to the area under the receiver operating characteristic curve (AUC). A *c*-statistic of 0.5 indicates that the model is no better than chance, and a value of 1 indicates that the model perfectly classifies the individuals.

### Statistical analysis: sensitivity analyses

Sensitivity analyses were performed to evaluate assumptions made during the main analyses.

The presence of an interaction between pain intensity rating and treatment arm was tested in the point of care and questionnaire models by including an interaction term of pain intensity rating with treatment arm (POST data only), as responders who received treatment may have a different relationship between their pain ratings and outcome than those who did not receive treatment.

Our main analyses only included patients with complete data for point of care, questionnaire and outcome. To evaluate the impact of including other patients with some missing data, the main analyses were repeated, first by including those extra patients in the point of care model who had missing information at the questionnaire and then by including the extra patients in the questionnaire model who had missing information at the point of care.

All analyses were performed using Stata/MP 14.0 (Stata Corporation, TX, USA).

## Results

### Data description

**Fig. 1:**
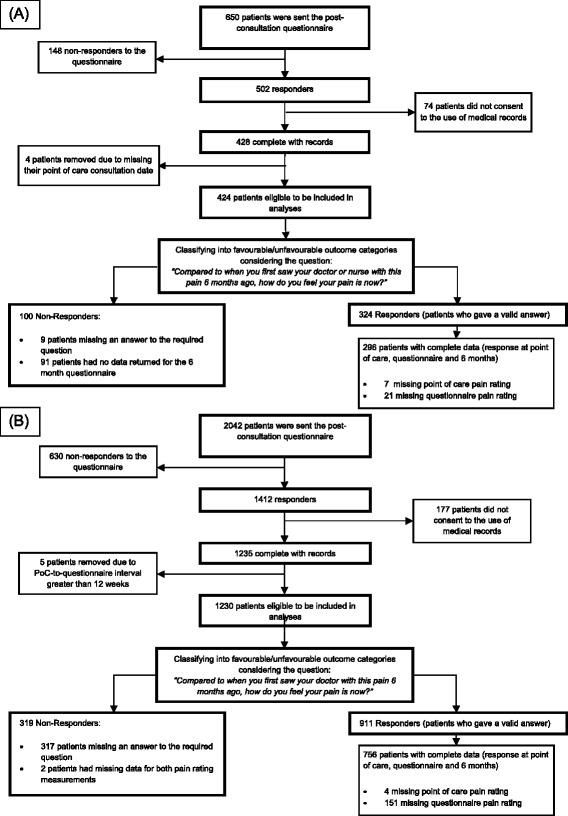
Participant flow. **a** PROG-RES. **b** POST

Of 650 potentially eligible patients mailed a questionnaire in PROG-RES, 424 (65.2%) returned it, consented to medical record review and had information at their consultation recorded, of whom 296 (45.5%) were also successfully followed up at 6 months. The corresponding figures for POST were 2042, 1230 (60.2%) and 756 (37.0%) (flowcharts provided in Fig. 1). Data was complete for age, gender, general practice and (in POST) treatment arm. Potentially eligible patients lost to follow-up at 6 months did not differ by age or gender but had slightly higher mean pain ratings at the point of care and in the questionnaire in PROG-RES (point of care: mean (SD): responders 5.9 (2.2) vs non-responders 6.4 (2.2); questionnaire: 5.5 (2.6) vs 5.6 (2.5)) and in POST (6.2 (2.1) vs 6.6 (2.0); 5.3 (2.6) vs 5.8 (2.6)).

Table 1 shows the characteristics of those with complete data included in the main analyses. The proportion reporting an unfavourable outcome at 6 months was 48.7% in PROG-RES and 54.5% in POST. In both studies, a significant fall in pain intensity ratings between point of care and the questionnaire measurement was observed, tested using a paired *t* test (PROG-RES: mean (SD) 5.9 (2.2) vs 5.5 (2.6), mean difference (SD) 0.42 (0.17), *P* = 0.006; POST: 6.2 (2.1) vs 5.3 (2.6), 0.89 (0.09), *P* < 0.001).

### Preliminary analyses

In PROG-RES, a significant mean reduction in pain score overall between point of care and questionnaire was observed in the group who went on to experience a favourable outcome at 6 months (mean reduction (SD) 1.12 (0.24), *P* < 0.001) but not in those with an unfavourable outcome (−0.32 (0.21), *P* = 0.932). Similar mean reductions were seen in POST (favourable outcome 1.59 (0.15), *P* < 0.001; unfavourable outcome 0.31 (0.12), *P* = 0.004).

### Examination of predictor-outcome associations

**Fig. 2:**
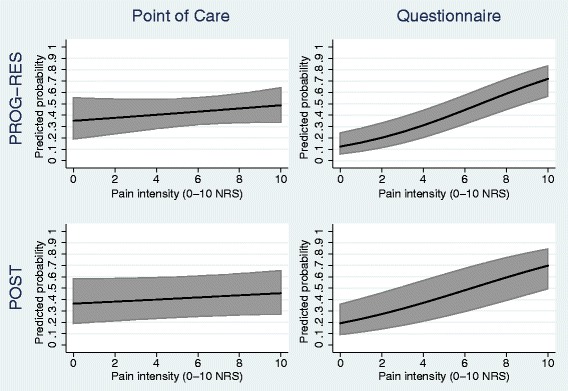
Predicted probability (95% confidence interval) of an unfavourable outcome at 6 months by pain intensity rating estimated from the point of care and questionnaire models (for a female patient from a randomly selected practice with the mean age in the dataset)

At the point of care, there was only a weak and non-statistically significant independent association found between pain intensity and an unfavourable outcome in both PROG-RES (adjusted OR (95% CI) 1.06 (0.95, 1.18)) and POST (1.04 (0.96, 1.12)) (Table 2). To translate this to absolute risk, we transformed the fitted models back to the probability scale. Figure 2 shows that (for a female patient from a randomly selected practice with the mean age in the dataset) there was little change in the predicted probability of an unfavourable outcome as pain intensity at point of care increased, in both POST and PROG-RES.

**Table 2:** Predictor-outcome association between a one-unit increase in pain intensity and an unfavourable outcome

	Intended moment of using the prognostic results	PROG-RES (*n* = 296)	POST (*n* = 756)
		Adjusted OR^a^ (95% CI)	Adjusted OR^a^ (95% CI)
Current pain intensity (0–10 NRS) measured at:			
Point of care	Yes	1.06 (0.95, 1.18)	1.04 (0.96, 1.12)
Post-consultation questionnaire	No	1.34 (1.20, 1.48)	1.26 (1.18, 1.34)

^a^Adjusted for age, gender, general practice (and treatment allocation—POST only)

In contrast, the models estimating the independent association between the questionnaire pain rating and outcome found a stronger and statistically significant relationship. In PROG-RES, for each unit increase in pain rating, the odds of an unfavourable outcome increased by 34% (adjusted OR (95% CI) 1.34 (1.20, 1.48)), and in POST, for each unit increase in pain rating, the odds of an unfavourable outcome increased by 26% (1.26 (1.18, 1.34)) (Table 2). Transforming the models back to the absolute risk scale, Fig. 2 shows that (for a female patient from a randomly selected practice with the mean age in the dataset) the predicted probability of an unfavourable outcome increased at similar rates as pain intensity at the questionnaire increased, in both datasets. The change in predicted probability is far steeper for the questionnaire models than for the point of care models. For example, in POST, the predicted probability for an individual with a pain score of 8 was 0.59 when using the questionnaire model but 0.44 when using the point of care model.

### Examination of prognostic model performance

Table 3 shows the performance measures for the fitted models from Table 2. The AIC for the questionnaire models was lower than that for the point of care models in both datasets, with a difference of 32 units in PROG-RES and 50 units in POST, suggesting that the models fitted using the pain score measured in the questionnaire had a better overall fit than the models using the pain score recorded at the point of care. The *c*-statistics were higher for the questionnaire models than for the point of care models in both datasets, and thus, the discrimination was larger when pain intensity was measured in the questionnaire. This concurs with the larger odds ratio estimates for pain intensity from the questionnaire than those from the point of care.

**Table 3:** Measures of model performance at the point of care and questionnaire in PROG-RES and POST

	Intended moment of using the prognostic results	PROG-RES		POST	
		AIC	*c*-statistic	AIC	*c*-statistic
Current pain intensity (0–10 NRS) measured at:					
Point of care	Yes	421.8	0.57 (0.51, 0.64)	1066.2	0.66 (0.62, 0.70)
Post-consultation questionnaire	No	389.8	0.69 (0.63, 0.75)	1015.8	0.72 (0.68, 0.76)

### Sensitivity analyses

We found no strong evidence of an interaction between treatment arm and pain intensity ratings at the point of care (OR (95%CI) 0.92 (0.78, 1.08)) or between treatment arm and questionnaire pain ratings (0.92 (0.81, 1.06)) in the POST dataset.

In the sensitivity analyses deriving models including the patients missing pain ratings either at the point of care or at questionnaire, the strength of associations between pain intensity and outcome did not change from those found in the main analyses: PROG-RES point of care: OR (95% CI) 1.06 (0.96, 1.18), *n* = 303; questionnaire: 1.34 (1.20, 1.48), *n* = 317 and POST 1.04 (0.96, 1.12), *n* = 757; 1.24 (1.17, 1.31), *n* = 904.

## Discussion

Our study illustrates how the magnitude of predictor-outcome associations and prognostic model performance can depend on *when* and/or *how* time-varying predictors are measured. In our example with patients presenting with musculoskeletal pain to general practice, associations between outcome risk and pain intensity recorded at the intended moment of use were lower in magnitude than those associations derived from a self-complete questionnaire mailed to patients up to 1 week later. Our findings were replicated in two datasets. Despite many published studies of musculoskeletal pain in primary care [20], very few report the collection of time-varying predictor information by the GP at the initial point of care [26]. When a later time is used, and/or with a different measurement method, the study’s predictor-outcome associations and prognostic model performance may be misleading, and thus, it could signal that the study is at high risk of bias and not applicable for its intended purpose.

Several phenomena may contribute to the observed discrepancy in predictor-outcome associations at the point of care and at a later time point. Firstly, the timing of predictor measurement may be critical. For example, most musculoskeletal disorders follow an episodic course and therefore, as would be expected, patients in POST and PROG-RES were likely to consult when their pain was more severe than usual. This creates the conditions for regression to the mean following the point of care [27, 28]. An initial reduction in group-average pain intensity rating within the first few days following primary care consultation has been consistently observed for acute, recurrent and chronic low back pain [29–32]. A similar pattern is likely across other non-inflammatory regional musculoskeletal pains. Although regression to the mean was evident within this study, the whole group mean was lower at the post-consultation questionnaire than at the point of care and so regression to the mean does not, therefore, provide a full explanation for the findings.

The differences found in the strengths of the predictor-outcome associations could also relate to differences in measurement methods. At the point of care, pain intensity measurement was verbally administered and recorded by the physician in a face-to-face consultation. Although in both studies physicians were given guidance on how to gather this information, we cannot know the extent to which physicians recorded their judgements of patients’ pain. Physician ratings tend to systematically underestimate patients’ own ratings of pain [33, 34]. Assuming that patients’ pain ratings were elicited and faithfully recorded at the point of care, it is nevertheless possible that a form of end-aversion bias [15] may operate in the clinical encounter, i.e. patients avoid reporting pain at either end of the severity scale in fear of being judged undeserving or exaggerating (although evidence from this study suggests this may be true of the lower end of the scale but not of the upper end of the scale).

A related issue is measurement error, such that—even if the setting and method of measurement were consistent—the predictor-outcome associations may not agree simply by chance variation. Further, if the measurement error was largest at the point of care, then the observed predictor-outcome association may be more biased at the point of care, than observed when measured at a later time point. If measurement error is present, it is likely that in this situation it would be differential measurement error, and the impact of differential measurement could either exaggerate or underestimate the effect. Indeed, the predictor-outcome associations estimated in this study at the point of care and at questionnaire are both likely to be biased as we did not adjust for measurement error due to insufficient information. Nevertheless, this is unlikely to account for the entire difference in magnitude of the estimated associations at point of care and questionnaire. Dependent error is also likely within this example, as a reduction in pain after the consultation (measured in the post-consultation questionnaire) is intrinsically going to be part of the patient’s judgement at 6 months about whether or not they have improved, particularly because these were measured by the same method, and this bias will likely be greater the closer in time the post-consultation questionnaire measurement is to the measurement of the outcome. This is a limitation of this particular example, and the bias created by this limitation may be less likely to be encountered in other prognostic models.

We focussed on predictor-outcome associations intended to be used at the point of care but derived using data collected after the point of care. It may be that a review appointment 2–3 weeks after the first consultation may be a better “intended moment of use” for prognostic models in this field. Either way, it is clear from our example that the developed prognostic model needs to use data for time-varying predictors measured at the time of its intended use, as otherwise discrepant associations may be included. It may be considered that the model using the score at the later time point should be used as this is performing better, but this model would be misleading if used during the consultation. For example, if we look at the example prediction plots in Fig. 2, if a patient visited their GP and reported a pain intensity score of 8, using the model developed with the score from the questionnaire would give this patient a predicted probability of experiencing an unfavourable outcome of 0.65. If the model developed using the point of care score was used, their predicted probability would be approximately 0.5.

While we believe that the problem we highlight may extend to other commonly investigated predictors whose values are sensitive to the timing and mode of collection, we have only demonstrated this problem for one predictor and thus this remains to be evaluated more widely. Further research should assess whether similar findings are found with other time-varying predictors and indeed in other clinical conditions and settings.

A future study in which the same mode of data collection is used at the point of care and at post-consultation questionnaire (e.g. patient self-administered questionnaire) is needed to better understand the relative contribution of timing and mode of collection and therefore determine whether and how improved prediction is achievable at the point of care.

## Conclusions

Our findings imply the need for caution when applying predictor-outcome associations or prognostic models derived from prognosis research studies that record time-varying predictors at a different time and/or by a different measurement method than is intended upon clinical application. This argument reinforces the need for clearly reporting the intended moment of use in prognostic research and when the predictors were measured [6]. Displacing the collection of time-varying predictor information from the intended moment (and mode) of use can result in differences in the magnitude of predictor-outcome associations and the subsequent accuracy of prognostic model performance. In particular, predictors and models that appear to discriminate well in research studies may fail to live up to those expectations when applied or externally validated at the intended moment of use. This concern is likely to be particularly justified when the outcome in some way incorporates the prognostic factor, when the interval between later measurement and outcome is short, and when the same mode of assessment is used to collect predictor and outcome information [35]. Unless shown otherwise in validation studies using predictors measured at the correct time, previously developed prediction models that include time-varying predictors measured after the intended moment of use may overestimate individual risk of experiencing the outcome of interest, which also reinforces the need for external validation and reporting of differences between validation and development data [5].

## Abbreviations

AIC: Akaike’s information criterion
AUC: Area under the receiver operating characteristic curve
CI: Confidence interval
GP: General practitioner
IQR: Inter-quartile range
NRS: Numerical rating scale
OR: Odds ratio
PMR: Polymyalgia rheumatic
POST: Primary care Osteoarthritis Screening Trial
PROG-RES: Prognostic Research Study
RCT: Randomised controlled trial
SD: Standard deviation
SpA: Spondyloarthritis
TRIPOD: Transparent Reporting of a multivariable prediction model for Individual Prognosis or Diagnosis
